# Investigation of the Prediction Accuracy of a Finite Element Analysis Model for the Coating Thickness in Cross-Wedge Rolled Coaxial Hybrid Parts

**DOI:** 10.3390/ma12182969

**Published:** 2019-09-12

**Authors:** Arne Jagodzinski, Jens Kruse, Alexander Barroi, Maximilian Mildebrath, Jan Langner, Malte Stonis, Marius Lammers, Jörg Hermsdorf, Thomas Hassel, Bernd-Arno Behrens, Ludger Overmeyer

**Affiliations:** 1 Institut für Integrierte Produktion Hannover gGmbH (IPH), Hollerithallee 6, 30419 Hannover, Germany; kruse@iph-hannover.de (J.K.); langner@iph-hannover.de (J.L.); stonis@iph-hannover.de (M.S.); behrens@ifum.uni-hannover.de (B.-A.B.); 2 Laser Zentrum Hannover e.V. (LZH), Hollerithallee 8, 30419 Hannover, Germany; a.barroi@lzh.de (A.B.); m.lammers@lzh.de (M.L.); j.hermsdorf@lzh.de (J.H.); ludger.overmeyer@ita.uni-hannover.de (L.O.); 3 Institut für Werkstoffkunde, An der Universität 2, 30823 Garbsen, Germany; mildebrath@iw.uni-hannover.de (M.M.); hassel@iw.uni-hannover.de (T.H.)

**Keywords:** cross-wedge rolling, hybrid forming, FEA, coating thickness

## Abstract

The Collaborative Research Centre 1153 (CRC 1153) “Process chain for the production of hybrid high-performance components through tailored forming” aims to develop new process chains for the production of hybrid bulk components using joined semi-finished workpieces. The subproject B1 investigates the formability of hybrid parts using cross-wedge rolling. This study investigates the reduction of the coating thickness of coaxially arranged semi-finished hybrid parts through cross-wedge rolling. The investigated parts are made of two steels (1.0460 and 1.4718) via laser cladding with hot-wire. The rolling process is designed by finite element (FE)-simulations and later experimentally investigated. Research priorities include investigations of the difference in the coating thickness of the laser cladded 1.4718 before and after cross-wedge rolling depending on the wedge angle β, cross-section reduction ΔA, and the forming speed ν. Also, the simulations and the experimental trials are compared to verify the possibility of predicting the thickness via finite element analysis (FEA). The main finding was the ability to describe the forming behavior of coaxially arranged hybrid parts at a cross-section reduction of 20% using FEA. For a cross-section reduction of 70% the results showed a larger deviation between simulation and experimental trials. The deviations were between 0.8% and 26.2%.

## 1. Introduction

Most technical components like connecting rods or shafts are made of a single mono-material. On one hand this grants specific material characteristics and therefore predictable forming behavior. On the other hand, it limits the areas of application for these components since the demands on their weight and size but also on their endurance rises continuously. To meet these demands, the usage of hybrid parts—which consist of two or more different materials—is a promising approach.

At the same time, process specific challenges also rise when using hybrid parts made of two or more materials, since they have different mechanical properties. When using different steels in a forming process, the flow stresses of those materials are the most challenging property. Each of the materials has a different flow stress at the same temperature, leading to an uneven forming behavior [1].

In this study, the reduction of the coating thickness of coaxially arranged semi-finished workpieces through subsequent cross-wedge rolling (CWR) is investigated. The main goal of this investigation is to evaluate the possibility to predict the coating thickness of laser cladded hybrid parts via the means of FEA. The coating thickness is a critical factor for the resilience of functional surfaces in hybrid parts. The workpiece used for this investigation serves as an example for a load adjusted shaft with highly loaded functional surfaces for bearings or gearwheels. At the same time the workpiece consists of a minimum amount of expensive high-alloyed steel without having any disadvantages regarding the mechanical properties. For this purpose, a cylindrical steel part made of C22.8 (1.0460) was partially encased with a coating of X45CrSi9-3 (1.4718) via laser cladding with hot-wire. The hybrid part was then incrementally formed using CWR. The presented investigation focuses especially on the forming behavior of the laser cladded material during the CWR process and the possibilities to predict the thickness of the cladded coating after the deformation using finite element analysis (FEA). The simulated results are compared to and verified by experimental trials.

### 1.1. Cross-Wedge Rolling

Cross-wedge rolling is a preforming process used to produce rotationally symmetrical workpieces with unequal mass distribution. The forming takes place between two oppositely moving wedge-shaped tools. The wedges are used to distribute the material in an axial direction. The high material utilization of up to 100% that can be achieved by CWR is one of its main benefits and the reason for it to be a first-choice process for preforming.

**Figure 1:**
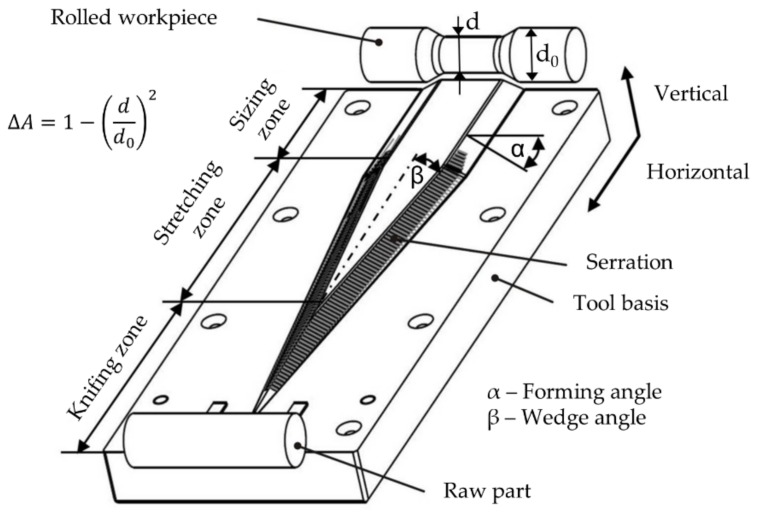
Principle of a cross-wedge rolling (CWR) process, including important forming parameters.

The principal layout of cross-wedge tools consists of three subsequent zones: in the knifing zone the wedge cuts into the workpiece, while the main forming takes place in the stretching zone. The diameter gets decreased and the workpiece is elongated. The last zone is the sizing zone. This is used to calibrate the workpiece and to roll out marks from the serrations on the shoulders of the wedges (Figure 1).

The main parameter used to describe a cross-wedge rolling process are the forming angle α and wedge angle β of the tool and the cross-section reduction ΔA. Other parameters that are important for the process are the billet and tool temperature, the forming speed ν, and the used billet materials.

A study on the process stability of cross-wedge rolling processes was performed by Pater et al. [2]. They were able to define process windows for stable CWR processes depending on the forming angle α, wedge angle β, and the relative reduction of the workpiece. These process limits were considered in the experimental design of the present paper.

Li and Lovell performed a study on the critical friction of a two-roll CWR process [3]. They described the critical friction as the friction which is required to establish rotation of the billet. They found that for the observed process, the critical friction lies between μ=0.2 and 0.3. They also found that an increase of the cross-section reduction ΔA and forming speed ν leads to an increase in global slip due to the decrease of the flow stress of the material. To ensure a rolling of the billets the friction factor was increased in the simulations performed in this study.

Pater showed the possibilities of tool optimizations in cross-wedge rolling [4]. He described a selection procedure for the forming angle α and wedge angle β, as well as ways to optimize the wedges shoulders. Since the influence of the wedge angle β is a main evaluation parameter, the described procedures were not applied in this study.

In an investigation by Li et al., the morphology of internal defects in cross wedge rolled workpieces were described [5]. They examined the generation and growth of defects like internal voids in 110 H16 aluminum (Al99.0Cu) billets depending on the forming angle α, wedge angle β, and the cross-section reduction ΔA. They also established a non-dimensional deformation coefficient which helps to predict the occurrence of internal voids. However, they only performed their studies with one aluminum alloy. Statements on the general applicability of their findings were not made.

Cross-wedge rolling is suitable for numerous materials. Studies by Çakırcalı et al. [6] and Li et al. [7] showed that it is possible to roll Ti6Al4V (3.7165) at temperatures between 500 °C and 950 °C. Blohm et al. investigated the simulation parameters for cross-wedge rolling Ti6Al4V (3.7165) and compared the simulated results to experimental trials performed with the same parameters [8]. They showed that the simulated forming forces are generally lower than in the experimental trials but the forming behavior in the trials is better than in the simulations. Wensheng et al. performed an experimental study on the cross-wedge rolling of a 6061 aluminum alloy (3.3211) [9]. They were able to show the feasibility of cross-wedge rolling a 6061 aluminum alloy at temperatures of 300–350 °C. Pater and Tomczak showed the general feasibility of cross-wedge rolling of numerous non-ferrous metal alloys [10]. They examined six different materials including aluminum (3.3206 and AlCu2Mg1.5Ni), titanium (3.7165), and magnesium alloys (Mg3Al1ZnMn, Mg6Al1ZnMn and Mg4AlZnMn). They were able to show the possibility of rolling all of the examined materials. However, none of these studies used hybrid parts composed of more than one material.

Rasche et al. investigated the use of cross-wedge rolled preforms for multi-directional forging [11]. They investigated the influence of the cross-section reduction in cross-wedge rolling on the multi-directional forging of crankshafts. They showed that a high cross-section reduction leads to a reduced flash on the crankshafts and better forming conditions in the multi-directional forging.

These studies only investigated CWR processes with monolithic materials. Hybrid parts made of two or more different materials were not part of the investigations.

### 1.2. Hybrid Parts

In sheet metal forming, tailored blanks are used as hybrid components. They mostly consist of two or more different sheet metals with different properties. Usually they differ in thickness, shape, strength, or materials that are welded together before forming. Assunção et al. performed a comparative study on laser welded tailored blanks in which they compared the performance of different laser types in terms of productivity, costs and welding quality [12]. They were able to weld samples with different sheet thicknesses and successfully form them without damaging the weld. Hybrid forged workpieces can be produced by combining sheet metal and bulk parts in one process step. This way lightweight components can be produced with cost and time savings. A novel hybrid forming process is tailored, forming where two or more materials are joined in one workpiece and are subsequently formed in a bulk forming process.

Peng et al. investigated the feasibility of cross-wedge rolling laminated shafts and analyzed the stress distribution during the rolling process [13]. They used S235JR (1.0038) as a base material laminated with 42CrMo4 (1.7225). They showed that it is possible to successfully reform a 1.7225/1.0038 laminated shaft via CWR. They also showed the significance of a proper selection of process parameters as the likelihood of a separation between the two materials increased with a poor parameter selection. Later they verified their findings in experimental trials [14]. They could show that the forming angle α has a smaller impact on the interface bonding then the wedge angle β. They found that with an increasing wedge angle β, the risk of a large ovalization of the cladding layer also increases. They were able to successfully roll a shaft made of S235JR (1.0038) laminated with 42CrMo4 (1.7225) at cross-section reductions up to 60%. However, the cladding was not welded onto the base material but instead was stacked over it.

Behrens et al. showed the general feasibility of cross-wedge rolling of coaxially arranged hybrid parts [15]. They performed experimental trials on workpieces made of 42CrMo4 (1.7225) coated with either 30MnCrTi4 (1.8401) or X45CrSi9-3 (1.4718). They were able to show that neither one of the coating materials peeled off of the base material. Furthermore, they showed that surface defects on the coating resulting from the welding process as well as pores on the inside have been closed by the forming process. They did not perform any investigations on the thickness of the coating material after the deformation.

A first investigation of the coating thickness of cross-wedge rolled hybrid parts was presented by Blohm et al. [16]. They showed that the main influences on the coating thickness after the forming process are the initial coating thickness and the cross-section reduction ΔA. But they only performed experimental trials and no simulations. Therefore, they did not make a statement on the possibilities to reliably predict the forming behavior and coating thickness of deposition welded coaxially hybrid parts using FEA.

An investigation on the predictability of the forming behavior of bulk hybrid parts and the deviation between simulation and experimental trials was performed by Blohm et al. in a case study [17]. They showed that for serially arranged hybrid parts, the deviation of the deformation of the joining zone between simulations and experimental trials is about 3%. However, they did not perform investigations on coaxially arranged hybrid parts.

None of these studies investigated the behavior of the coating thickness of laser cladded coaxially arranged hybrid parts during CWR.

## 2. Investigations of the Coating Thickness

**Figure 2:**
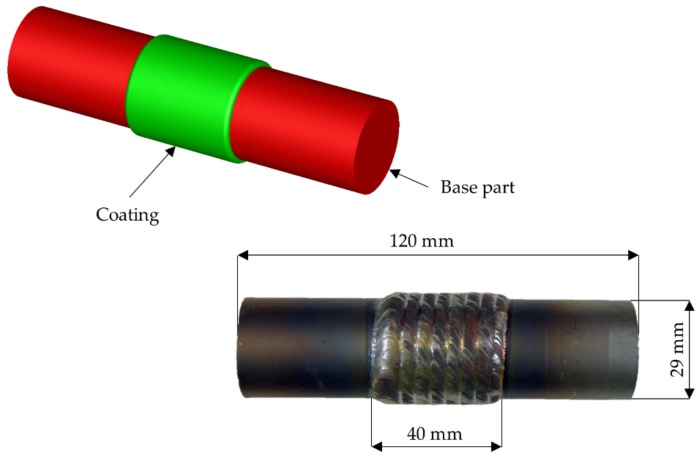
FEA-model (**above**) and real picture (**below**) of the geometry used for the investigations.

In this study, the coating thickness of laser cladded coaxially arranged hybrid parts after CWR is investigated. These investigations started by performing finite element (FE)-simulations followed by experimental trials. The geometry used for these investigations consisted of a cylindrical base part of C22.8 (1.0460) and a cladded coating of X45CrSi9-3 (1.4718) in the middle of the workpiece (Figure 2). The main input parameters of this study are the wedge angle β, cross-section reduction ΔA, and forming velocity ν, while the main output parameter is the change of the coating thickness during the rolling process. This will be used to determine the deviations between the coating thicknesses predicted by FE-simulations and those achieved in experimental trials and therefore the prediction accuracy of the used model.

**Figure 3:**
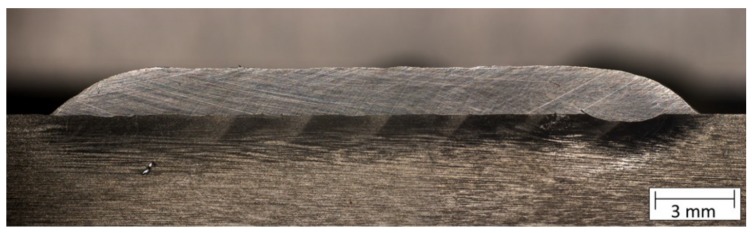
Cross-section of the welding coating with even surface and minimal dilution.

For the cladding, a scanner-supported laser hot-wire process has been used. This involves a Laserline diode laser, a 2-D laser scanner, and a DINSE hot-wire system as shown by Barroi et al. [18,19]. The cladding was performed as a helix, using a rotational axis which was moved on a linear stage. The advantage of this cladding geometry is that the process does not have to be interrupted, what leads to a smoother surface that rolls easier during CWR. For the cladding process, 1800 W of laser power which was scanned perpendicular to the welding direction and 105 A of electric current to heat the wire were used. The wire with a diameter of 0.8 mm was fed at 3.6 m/min. With the traverse speed on the surface of the billet of 310 mm/min a coating thickness of 1.8 mm was applied. Figure 3 depicts the very small dilution of the coating and the substrate material. This is an enormous advantage of the laser cladding process compared to other processes, since the alloy of the coating has not been changed.

The forming takes places in the area of the coating and reduces the diameter of the workpiece locally. The reduction of the diameter is described as the cross-section reduction, which is defined as the change of the initial cross-section $A_{0}$ compared to the cross-section after the forming $A_{1}$ (Equation (1)).

(1) $$\Delta A=\frac{A_{0}-A_{1}}{A_{0}}=1-{\left(\frac{d_{1}}{d_{0}}\right)}^{2}$$

During the experiments the main CWR process parameters wedge angle β, cross-section reduction ΔA and forming velocity ν were varied (compare Figure 1 and Table 1). The forming angle α was not changed during the experiments since it has no influence on the coating thickness, as was identified in pretrials. The initial coating thickness was also not changed during the investigations. Even though Blohm et al. identified the initial coating thickness as one of the main influences on the final coating thickness, further investigation was not found to be necessary, since the goal of this investigation is neither to determine certain coating thicknesses that can be reached with CWR nor to investigate the resilience of the coating [16]. The goal of this investigation is to determine the possibility of the prediction of the change in coating thickness via FEA and therefore the prediction accuracy of the used model. The influence of the parameters on the coating thickness of the cladded coating material were examined. The method design of experiments (DoE) were used for this examination. It allows us to set two or more different levels for each investigated parameter. When using two different levels, a low and a high level should be preferred. The results of the investigation were then analyzed using the statistically approved t-test to determine the influence of the parameters on the thickness.

The aim of the simulations is to get results as close to the experimental trials as possible. This is mandatory in order to be able to predict to behavior of the coating thickness using FE-simulations. Therefore, the deviation between simulations and trials shall be minimal. For this investigation, a target deviation of 10% is aimed for. This will be realized with a billet mesh size of 1 mm in the forming zone. Since neither end of the workpiece will be formed, the mesh size for these areas of the billet is set to 5 mm to reduce the time needed for the simulation. Also, the FEA-model of the billet was rebuilt as close to the real part as possible (Figure 2). 

Each process parameter used for the investigation except for the workpiece temperature consists of a high and low level (Table 1). The materials used were C22.8 (1.0460) for the base part and X45CrSi9-3 (1.4718) for the coating. The billets diameters were 29 mm for the base part and 32.6 mm for the coating.

### 2.1. Setup of the Simulations

**Figure 4:**
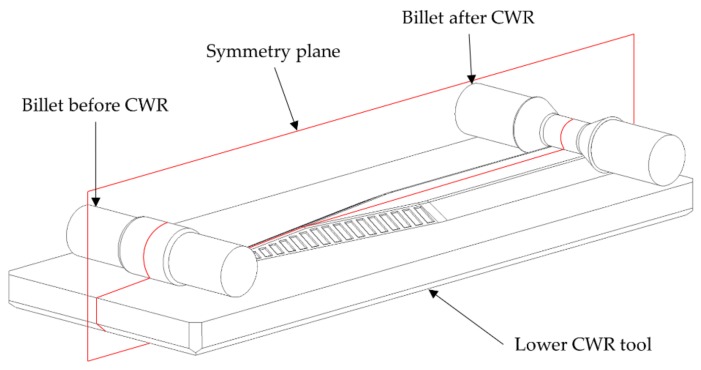
Model of the cross-wedge rolling process.

For the simulations of the present investigation the software FORGE NXT 2.1 was used. The CWR process is designed as a flat wedge process with two tools with the coaxially arranged hybrid semi-finished workpiece in the middle. To reduce the simulation time, the setup was mirrored on a symmetry plane (Figure 4).

**Figure 5:**
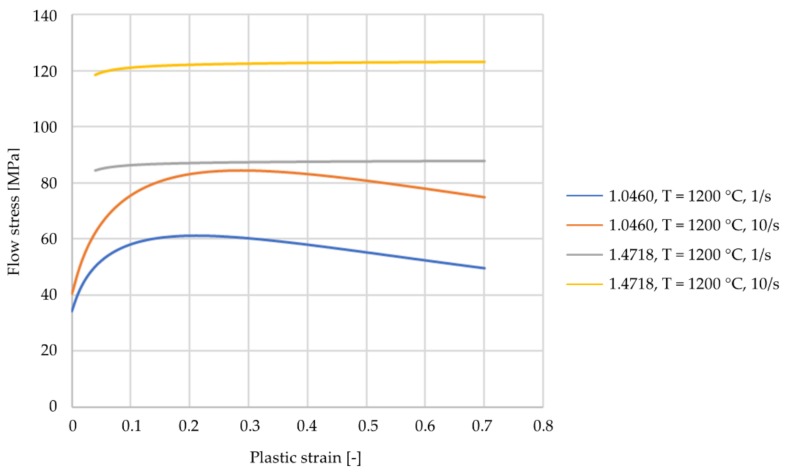
Flow curves for 1.0460 and 1.4718 in comparison.

The mechanical properties of the materials are the simplified standard values of the simulation program. They both have a density of $\rho =7850\frac{kg}{m^{3}}$, a specific heat of $c=778\frac{J}{kg\;K}$, a conductivity of $\kappa =35.5\frac{W}{m\;K}$, and an emissivity of $\varepsilon =0.88\frac{W}{m^{2}}\;$. However, the two materials differ in their flow curves. For the C22.8 (1.0460) the material description is based on the flow curves described by Behrens et al. [20]. The flow curves were recorded for the strain rates 1/s and 10/s at the temperatures of 600 °C, 750 °C, 900 °C, 1050 °C and 1200 °C. For other strain rates and temperatures, the values are interpolated or extrapolated. The material description for the X45CrSi9-3 (1.4718) is based on the flow curve equation by Hensel and Spittel. Their equation describes the forming behavior depending on the Temperature T, the effective strain and flow behavior ε and the strain rate $\dot{\varepsilon }$. FORGE NXT 2.1 uses a shortened version of the equation (Equation (2)). The necessary parameters are stored within the material database of the program. The parameters for X45CrSi9-3 (1.4718) are shown in Table 2. The flow curves of the two used materials are displayed and compared in Figure 5.

(2) $$\sigma_{f}=A\cdot e^{m_{1}T}\cdot e^{m_{2}}\cdot e^{\frac{m_{4}}{\varepsilon }}\cdot {\dot{\varepsilon }}^{m_{3}}$$

The bonding between the base material and the coating is realized via a bilateral-sticking-contact. This means the nodes of the mesh are fixed and cannot detach themselves from the surface of the contact partner. The thermal exchange between the base material and the coating is realized via a steel to steel contact with a transfer coefficient of $\alpha =10.000\;\frac{W}{m^{2}\cdot K}$. The transfer between the workpiece and the environment is set to a steel to air contact with a heat transfer coefficient of $\alpha \;=\;10\frac{W}{m^{2}\cdot K}$.

In CWR, a high friction is necessary for the workpiece to roll properly. In experimental trials, this is realized by adding serrations to the shoulders of the wedges. In simulations the friction factor needs to be increased. In regular hot forging simulations, a factor of m=0.3 is used, whereas in the present simulations it was increased to m=0.8 to ensure a rolling of the part.

### 2.2. Setup of the Experimental Trials

The objective of the experimental trials was the reduction of the coating thickness of coaxially arranged hybrid semi-finished parts using CWR. The experimental equipment consists of a hydraulic CWR module for flat wedge CWR tools. The module is compatible to and mounted on a hydraulic press of the manufacturer NEFF. The presses maximum forming force is 6300 kN. It is used to realize the roll gap between the tools and to put up the necessary locking force for the rolling process. The linear movement of the flat wedge tools is realized by two hydraulic cylinders with a maximum forming force of 125 kN each. They are connected to the wedge fixture by bearing blocks and move the forming tools. The CWR module allows forming speeds up to 240 mm/s. To avoid any effects resulting from an asymmetrical forming, the billets were centered in front of the wedge (Figure 4).

The trials were performed without lubrication to ensure the rolling of the billets. After a heating time of 30 min in an electric furnace they were manually handled. The hydraulic press was then closed with 500 kN to realize the locking force and the billets were rolled. After the forming the billets were cooled in air. For every parameter configuration two billets were cross-wedge rolled.

## 3. Results and Comparison of Simulation and Trials

After the CWR the billets from the experimental trials were cut in radial direction via wet grinding. The coating thickness was measured and compared to the corresponding simulations. The deviation of the coating thickness s after CWR between the simulations and the experimental trials is the main evaluation parameter. For this the coating thicknesses in the simulations and the experimental trials will be analyzed separately before comparing them.

### 3.1. Results of the Simulations

**Figure 6:**
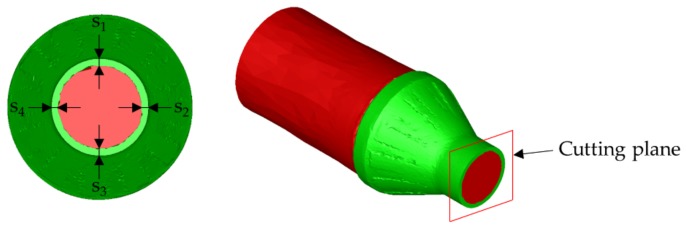
Measuring points in the simulations.

The measurement of the thickness was performed in four different places around the diameter of the workpiece (Figure 6). To have a single value for every simulated parameter combination the mean value for the four measured thicknesses is calculated. Additionally, the standard deviation σ for the thicknesses was determined as a measure for their homogeneity. All measuring points are located exactly in the lateral middle of the workpiece on the symmetry plane.

**Figure 7:**
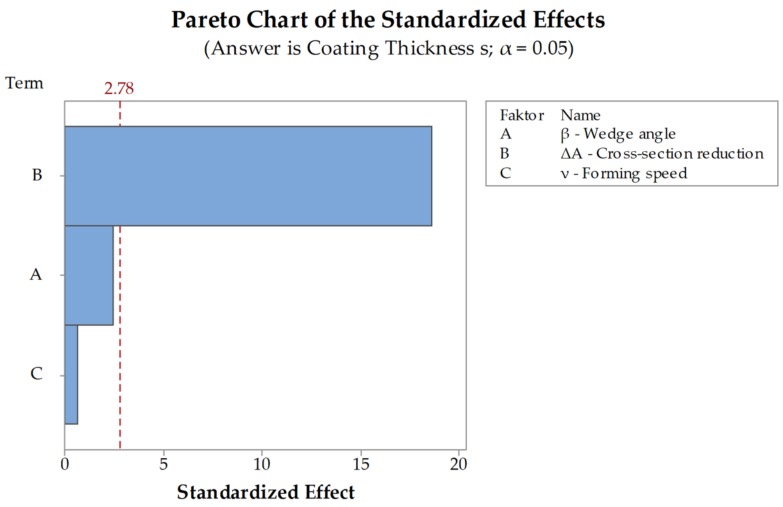
Pareto chart of the standardized effects for the coating thickness s in the simulations.

All workpieces showed a stable rolling behavior. The standard deviations for the simulations are between $\sigma_{min}=\;0.03\;mm$ and $\sigma_{max}\;=\;0.11\;mm$. The main differences in the coating thickness occur between the different cross-section reductions. At a reduction of ΔA=20 % the coating has an average thickness of s=1.44 mm while at a reduction of ΔA=70 % the average is about s = 0.7 mm. The decrease of the coating thickness at an increase of cross-section reduction can be explained with the increasing stresses that occur with an increasing cross-section reduction. The C22.8 (1.0460) has a lower flow stress than the X45CrSi9-3 (1.4718) and thus can be formed more easily (Figure 5). With an increasing cross-section reduction, the stress also increases leading to a forming of the X45CrSi9-3 (1.4718). Another explanation for this effect is the increasing material displacement at higher cross-section reductions. Differences depending on the forming speed and the wedge angle are minimal. These investigations are supported by the DoE-analyses performed for the simulations. The evaluation of the significance of the main parameters wedge angle β, cross-section reduction ΔA and forming speed ν shows that the only significant factor for the coating thickness after CWR is the cross-section reduction (Figure 7). The wedge angle β has no significant impact on the thickness nor has the forming speed ν. The average coating thicknesses after CWR for the investigated parameters are shown in Table 3.

**Figure 8:**
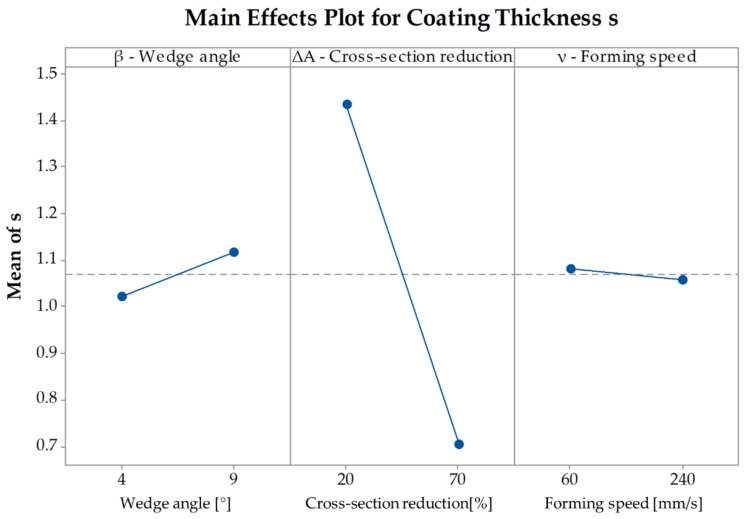
Main Effects Plot for the coating thickness s in the simulations.

To fully understand the effects of the parameters on the coating thickness it is necessary to examine the main effects plot as well. The pareto-chart indicates only if a parameter is significant or not, but it does not indicate absolute values of the influence of the parameters on the coating thickness. Figure 8 shows that with an increasing cross-section reduction the coating thickness decreases from s=1.44 mm at ΔA=20% to s=0.7 mm at ΔA=70%. An increase of the forming speed also leads to a decrease of the thickness. At a speed of ν=60 mm/s the average thickness is s=1.08 mm and at ν=240 mm/s the average thickness is s=1.06 mm. Since this parameter is not significant for the change of the coating thickness the difference between the two speeds is minimal. An increase of the wedge angle leads to an increase of the coating thickness but since the impact of the angle is not significant as well, the increase is also minimal with a coating thickness s=1.02 mm for β=4° and s=1.11 mm for β=9° (Figure 8). The values of the coating thickness s depicted in the main effects plot represent mean values over the parameters wedge angle, cross-section reduction, and forming speed.

**Figure 9:**
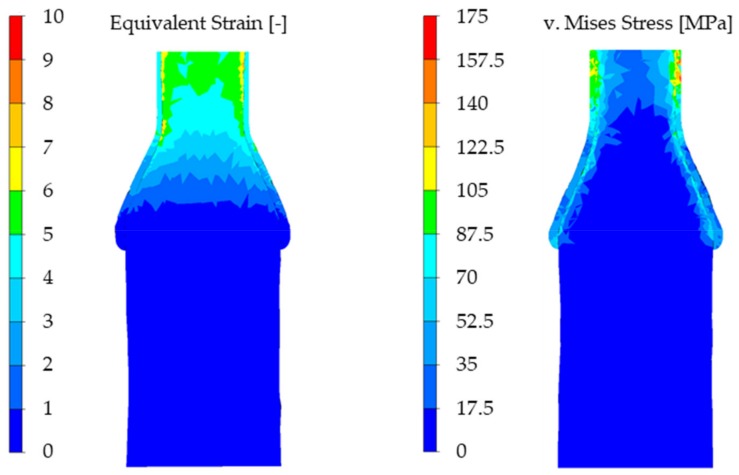
Equivalent strain and v. Mises stress shortly before the end of the forming process.

For every analyzed parameter configuration, the coating thickness decreased after CWR. The most obvious change took place at the cross-section reduction of ΔA=70% whereas the decrease at ΔA=20% reduction is less visible. The equivalent strain and the von Mises-Stress shortly before the forming process is finished are shown in Figure 9 exemplarily for the simulation with a wedge angle of β=9°, a cross-section reduction of ΔA=70%, and a forming speed of  ν=60 mm/s.

**Figure 10:**
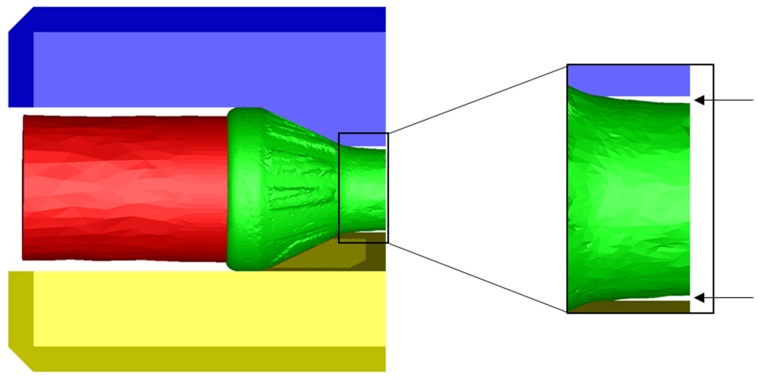
Detachment of the workpiece from the tool during the rolling process due to necking.

In the simulations, for a wedge angle of β=4° and a cross-section reduction of ΔA=70% the workpieces detached themselves from the tool surface due to a necking in the forming area (Figure 10). A possible explanation for this are friction parameters that are not optimally set. If they are set too high the friction on the wedge’s shoulders can lead to higher axial forces, which in turn can lead to a necking of the workpiece. In the corresponding simulations with a wedge angle of  β=9° this phenomenon was not observed. It is possible that this is due to the shorter tools at a larger wedge angle. On the longer tool with the wedge angle of  β=4°, the workpiece requires more rotations than on the shorter one, which can lead to a larger error in the calculation and thus to a necking at β=4° but not at β=9°.

### 3.2. Results of the Experimental Trials

**Figure 11:**
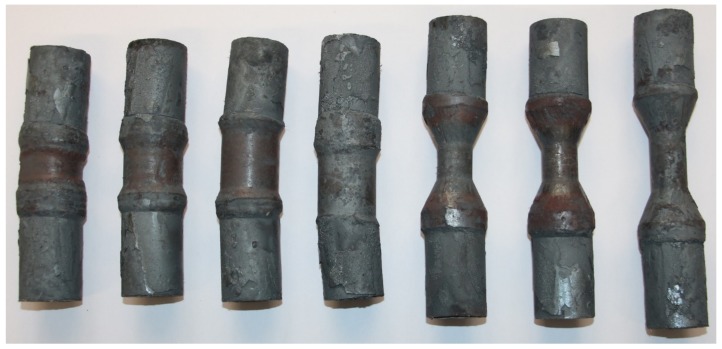
Workpieces after rolling.

The experimental trials showed a stable rolling behavior for all but one parameter configuration. For a wedge angle β=9°, cross-section reduction ΔA=70% and forming speed ν=60 mm/s, the workpiece began to slip shortly after the wedge started to cut into the material. This resulted in an oval cross section in the formed area of the workpiece. Figure 11 shows the workpieces that were not cut for further investigation. 

**Figure 12:**
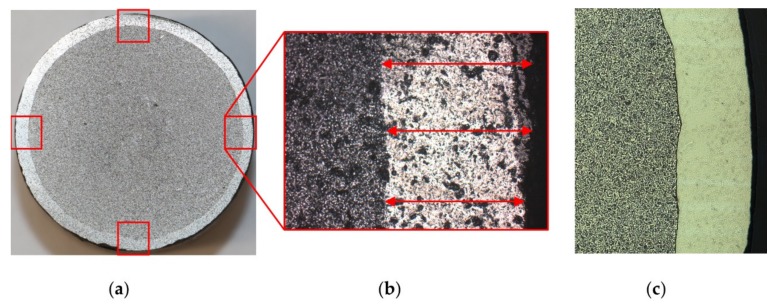
(**a**) Laser cladded coating after CWR, grinding and sandblasting with measuring points; (**b**) Picture of the coating after CWR taken by the microscopes camera; (**c**) Polished surface of the cut.

The measuring and analysis were performed with the use of a microscope equipped with a camera. The microscope was used to take four pictures of the coating after CWR around the diameter of the probes (Figure 12b). Afterwards the thickness was measured with the image processing software ImageJ. This was realized with a reference picture of 1 mm scale that was taken with the exact same microscope settings as the rest of the pictures. The software was used to measure the pixel/mm in the reference picture. Afterwards, three measurements were taken for every picture of the coatings (Figure 12, red arrows). For every workpiece, four pictures were taken (Figure 12, red squares) and the mean value for all twelve measurements were calculated. To simplify the measuring, for every analyzed workpiece a slice of the formed area was cut out. Since the simulations showed that the thickness of the cladding is uniform along the width of the formed area, it was assumed that this also applies to the experimental trials and that it is therefore acceptable to make the cut outside of the lateral middle of the workpiece. Due to the used materials the coating is clearly visible after sandblasting even without etching the grinded surface (Figure 12a). The material is free of pores (Figure 12c). It is very homogenous in the base material and shows no heat affected zone due to the heating and rolling in the CWR-process. This is one of the main advantages of a process chain which has the welding process take place before the forging process.

Just like in the simulation, the coating thickness varies most between the two different cross-section reductions. At a reduction of ΔA=20% the average coating thickness is s=1.34 mm while it amounts to s=0.84 mm for a cross-section reduction ΔA=70%. As already described, this can be explained with the increasing stresses as well as the increasing material displacement. The variation of the wedge angle β and the forming speed ν has almost no visible effect on the coating thickness after CWR like the simulations already shown. The standard deviations for the experimental trials lie between $\sigma_{min}=0.07\;mm$ and $\sigma_{max}=0.24\;mm$. The average coating thicknesses after CWR for the investigated parameters are shown in Table 4.

**Figure 13:**
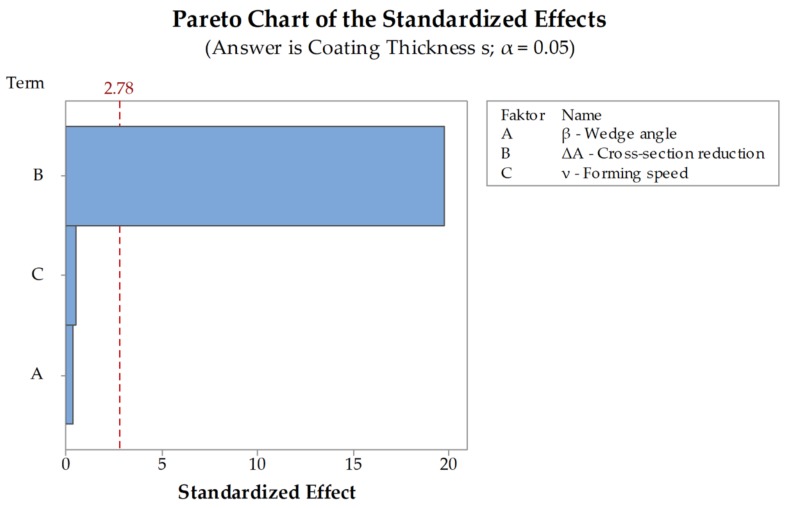
Pareto Chart of the Standardized Effects for the coating thickness s in the experimental trials.

The evaluation of the significance of the investigated parameters wedge angle β, cross-section reduction ΔA and forming speed ν performed via DoE-analysis showed that only the cross-section reduction ΔA has a significant impact on the coating thickness after CWR. Neither the wedge angle β nor the forming speed ν showed any significant behavior (Figure 13). It is noticeable that the significances of the factors A and C are transposed in comparison to the analysis of the simulations (Figure 7). Factors A and C are below the significance threshold, meaning their influences are small compared to factor B. Additionally, the values of the factors are very similar to each other, e.g., a small change in the measurement of the result, can result in a big change of the effect of a factor. In this case, the change in the factors probably occurred due to general inaccuracies of the FEA since it is based on an approximation method, that are already big enough to change the order of factor A and C.

**Figure 14:**
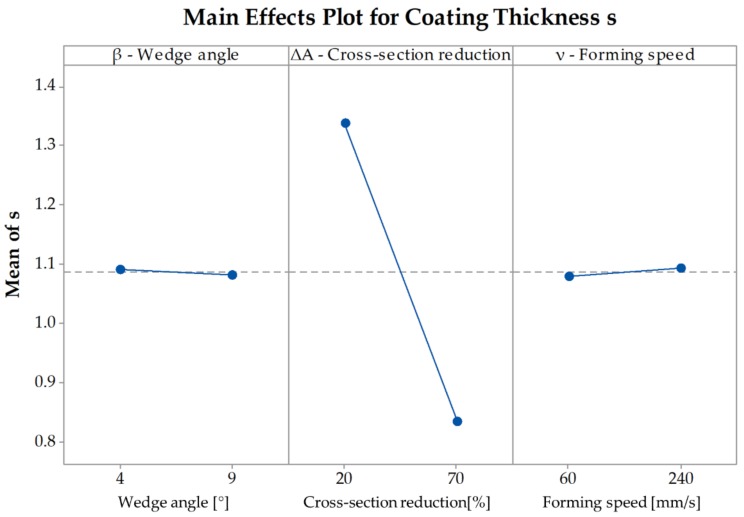
Main Effects Plot for the coating thickness s in the experimental trials.

To be able to determine the way the coating thickness changes with a change of the investigated parameters it is necessary to examine the main effects plot (Figure 14). The plot shows that with an increasing cross-section reduction the coating thickness decreases from s=1.34 mm at ΔA=20% to s=0.84 mm at ΔA=70%. Since the wedge angle and the forming speed are not significant to the coating thickness after CWR, there is only a minimal difference between β=4° and β=9° as well as between ν=60 mm/s and ν=240 mm/s.

For every analyzed parameter configuration, the coating thickness decreased after CWR. The most obvious change took place at the cross-section reduction of ΔA=70%, whereas the decrease at ΔA=20% reduction is less visible.

### 3.3. Comparison between the Simulations and the Experimental Trials

After the simulations and subsequent experimental trials, the results were compared to each other. Table 5 shows the comparison of the average coating thicknesses after CWR for every investigated parameter configuration between the simulations and the experimental trials. The deviation between the results is also shown. The results from the simulations and experimental trials with a cross-section reduction of ΔA=20% show satisfying deviations between 0.8% and 10.52%. For a cross-section reduction of ΔA=70% the deviations are between 5.11% and 26.24%. Especially the simulations with a wedge angle of β=4° and a cross-section reduction of ΔA=70 % show large deviations between the simulation and the experimental trials. A possible explanation for these large deviations is the necking of the workpiece in the middle of the forming area during these simulations. This leads to a stretching of the material in the necking area and therefore to an additional thinning of the cladding. Such behavior was not observed during the experimental trials, as no necking occurred in the workpiece centers for the regarding parameter configurations (Figure 11).

In order to exclude the possibility that the large deviations are caused by the simulation model used, a further simulation with a wedge angle of β=4°, a cross-section reduction of ΔA=70% and a forming speed of ν=240 mm/s was set up. For this the FEM software Forge NxT 3.0 was used. This includes multi-material sets with an improved remeshing, which improves the simulation of hybrid components with high degrees of forming. However, this simulation showed the same necking in the middle of the workpiece and the coating thickness was also at s=0.628 mm. 

## 4. Discussion

This paper discusses a basic investigation of the coating thickness of laser cladded coaxially arranged billets after CWR and the possibilities to use FEA for the prediction. The investigation was separated in two stages. In the first stage FE-simulations were performed followed by experimental trials in the second stage. The influence of the main CWR-parameters wedge angle β, cross-section reduction ΔA and forming speed ν on the thickness after rolling were studied and the result from the simulations and experimental trials were compared.

The coaxially arranged hybrids parts could be rolled without separation or destruction of the parts. The main influencing parameter for the coating thickness after CWR is the cross-section reduction ΔA. The results of the simulations and experimental trials showed that it is possible to reliably predict the change of coating thickness during the CWR process using FEA most investigated parameter configurations with a deviation between 0.83% and 10.52%. For β=4° and ΔA=70% however, it was not possible to predict the coating thickness with a satisfying accuracy—the deviations are between 23.85% and 26.24%.

The presented study only investigated the behavior of coaxially arranged semi-finished hybrid parts with a laser cladded coating during cross wedge rolling. The study does not include possibilities to control the forming behavior of the coating. Also, the influence of the laser cladding parameters was not investigated in the presented study. In order to understand why the large deviations occur only for a wedge angle of β=4° and a cross-section reduction of ΔA=70%, further investigations are necessary.

## Figures and Tables

**Table 1:** Process parameters of the CWR process.

Billet Material	Billet Dimension	Tool Geometry	Cross-Section Reduction ΔA	Forming Speed ν	Workpiece Temperature $T_{w}$
1.0460 + 1.4718	Ø 29/32.6 mm length 120 mm	α= 25° β= 4°–9°	20–70%	60–240 mm/s	1250 °C

**Table 2:** Values for the parameters of the Hensel Spittel equation for X45CrSi9-3 (1.4718).

A	$m_{1}$	$m_{2}$	$m_{3}$	$m_{4}$
552.26996	−0.00153	0.00339	−0.14771	−0.00131

**Table 3:** Average coating thicknesses after CWR for different process parameters in the simulations.

ΔA=20%	ΔA=20% ν=60 mm/s	ΔA=20% ν=240 mm/s	ΔA=20% β=4°	ΔA=20% β=9°
1.44 mm	1.46 mm	1.41 mm	1.41 mm	1.46 mm
ΔA=70%	ΔA=70% ν=60 mm/s	ΔA=70% ν=240 mm/s	ΔA=70% β=4°	ΔA=70% β=9°
0.70 mm	0.70 mm	0.71 mm	0.63 mm	0.78 mm

**Table 4:** Average coating thicknesses after CWR for different process parameters in the experimental trials.

ΔA=20%	ΔA=20% ν=60 mm/s	ΔA=20% ν=240 mm/s	ΔA=20% β=4°	ΔA=20% β=9°
1.34 mm	1.32 mm	1.36 mm	1.34 mm	1.33 mm
ΔA=70%	ΔA=70% ν=60 mm/s	ΔA=70% ν=240 mm/s	ΔA=70% β=4°	ΔA=70% β=9°
0.84 mm	0.84 mm	0.83 mm	0.84 mm	0.83 mm

**Table 5:** Comparison of the average coating thicknesses after CWR between the simulations and the experimental trials as well as the deviation between simulation and experimental trial.

β	ΔA	ν	s		Deviation	
			Simulation	Experiment	Absolute	Relative
4	20	60	1.467 mm	1.313 mm	0.154 mm	10.52%
4	20	240	1.361 mm	1.373 mm	0.011 mm	0.83%
4	70	60	0.603 mm	0.818 mm	0.215 mm	26.24%
4	70	240	0.657 mm	0.863 mm	0.206 mm	23.85%
9	20	60	1.459 mm	1.325 mm	0.134 mm	9.19%
9	20	240	1.454 mm	1.341 mm	0.113 mm	7.74%
9	70	60	0.8 mm	0.868 mm	0.068 mm	7.88%
9	70	240	0.757 mm	0.797 mm	0.041 mm	5.11%
